# Assessing Unique Risk Factors for COVID-19 Complications Among Cancer Patients: A Multi-ethnic Cohort Study

**DOI:** 10.1007/s10903-022-01413-w

**Published:** 2022-11-07

**Authors:** Hala T. Borno, Mi-Ok Kim, Irina Tolstykh, Amy Lin, Julian C. Hong, Sasha Yousefi, Sylvia Zhang, Rana R. McKay, Olivier Harismendy, Pedram Razavi, Pelin Cinar, Hope Rugo, Vadim S. Koshkin, Maya Rabow, Christine Wang, Adina Bailey, Eric J. Small

**Affiliations:** 1 Division of Hematology/Oncology, Department of Medicine, University of California, San Francisco, USA; 2 Helen Diller Family Comprehensive Cancer Center, San Francisco, CA USA; 3 Department of Epidemiology and Biostatistics, University of California, San Francisco, USA; 4 Department of Radiation Oncology, University of California, San Francisco, USA; 5 Bakar Computational Health Sciences Institute, University of California, San Francisco, USA; 6 Department of Medicine, University of California, San Diego, USA; 7 Moores Cancer Center, University of California, San Diego, USA; 8 College of Science, Northeastern University, Boston, MA USA

**Keywords:** COVID-19, Cancer, Racial/ethnic minorities

## Abstract

A myriad of organ-specific complications have been observed with COVID-19. While racial/ethnic minorities have been disproportionately burdened by this disease, our understanding of the unique risk factors for complications among a diverse population of cancer patients remains limited. This is a multi-institutional, multi-ethnic cohort study evaluating COVID-19 complications among cancer patients. Patients with an invasive cancer diagnosis and confirmed SARS-CoV-2 infection were identified from March to November 2020. Demographic and clinical data were obtained and a multivariate logistic regression was employed to evaluate the impact of demographic and clinical factors on COVID-19 complications. The study endpoints were evaluated independently and included any complication, sepsis, pulmonary complications and cardiac complications. A total of 303 patients were evaluated, of whom 48% were male, 79% had solid tumors, and 42% were Hispanic/Latinx (Hispanic). Malignant hematologic cancers were associated with a higher risk of sepsis (OR 3.93 (95% CI 1.58–9.81)). Male patients had a higher risk of sepsis (OR 4.42 (95% CI 1.63–11.96)) and cardiac complications (OR 2.02 (95% CI 1.05–3.89)). Hispanic patients had a higher odds of any complication (OR 2.31 (95% CI 1.18–4.51)) and other race was associated with a higher odds of cardiac complications (OR 2.41 (95% CI 1.01–5.73)). Clinically, fever, cough, and ≥2 co-morbidities were independently significantly associated with any complication. This analysis evaluated covariates that can significantly predict a myriad of complications among a multi-ethnic cohort of cancer patients. The conclusions drawn from this analysis elucidate a mechanistic understanding of differential illness severity from COVID-19.

## Introduction

In the United States, the current COVID-19 pandemic has disproportionately affected minority communities [1]. Despite this observation, racial/ethnic minorities remain underrepresented in published COVID-19 clinical research studies [2, 3]. Research to date has shown that certain demographic factors, such as race/ethnicity, have an impact on clinical severity and outcomes [4–8]. Among the general population infected with SARS-CoV-2, Hispanic/Latinx (Hispanic) ethnicity was observed to be strongly associated with a need for mechanical ventilation [9], whereas non-white ethnicity is strongly associated with critical care admission [8]. However, the impact of race and ethnicity in a cancer specific population remains underexamined. Patients with cancer may be particularly vulnerable to the morbidity and mortality associated with COVID-19 disease given underlying co-morbidities and potential immunosuppression from their disease and its treatment.

Individuals with an invasive cancer diagnosis and COVID-19 have been found to have an increased risk of all-cause mortality [10]. Given that COVID-19 can manifest with a myriad of complications, this study leverages a multi-institutional, multi-ethnic cohort to identify the unique risk factors for COVID-19 complications among a diverse population of patients with cancer.

## Methods

This is a multi-institutional, multi-ethnic cohort study among patients with cancer. Patients with an invasive cancer diagnosis and a positive serologic or molecular SARS-CoV-2 test result were identified at participating sites (the University of California San Francisco (UCSF), and the University of California San Diego (UCSD)). Demographic and clinical data were collected manually from the electronic health record from March 1 to November 30, 2020, and populated in a REDCap database [11, 12]. This study was deemed exempt from review by the UCSF institutional review board (IRB) and received local IRB approval at UCSD.

### Outcomes

We evaluated demographic, disease and treatment factors for their effects on four independent primary endpoints attributed to a COVID-19 diagnosis: “any complication”, sepsis, pulmonary complications, and cardiac complications were identified through manual chart review. Pulmonary complications were defined as respiratory failure, pneumonitis, acute respiratory distress syndrome (ARDS), pulmonary embolism, pleural effusion, or empyema. Cardiovascular complications were defined as hypotension, myocardial infarction, other cardiac ischemia, atrial fibrillation, ventricular fibrillation, other cardiac arrhythmia, cardiomyopathy, congestive heart failure, deep venous thrombosis, superficial venous thrombosis, cerebrovascular accident, or thrombosis. Patients with documented septicemia in the electronic health record were denoted as sepsis events. Lastly “other” complications were collected including gastrointestinal, bleeding, neurologic, systemic, or renal. Patients with “other”, sepsis, pulmonary complications, or cardiac complications were reported as “any complication”. Severity of COVID-19 outcomes for this study cohort defined as outpatient, hospitalization (non-ICU), ICU admission, and mortality is reported separately [13].

### Statistical Analysis

Descriptive statistics were employed to summarize the baseline demographic and clinical characteristics of the study cohort. The frequency of clinical presentation characteristics were stratified by COVID-19 complications. A multivariate logistic regression model was generated to evaluate the relationship between patient-level covariates such as cancer history, cancer status, sex, race, age, BMI, treatment history, co-morbidity and COVID-19 presentation. The number of co-morbidities was calculated through manual chart review. The odds ratio for the independent primary endpoints were determined. Facility and environmental factors were controlled for in the model as splines by including average number of COVID-19 cases in the last 7 days per facility.

## Results

### Patient Characteristics

The study cohort included 303 patients with an invasive cancer diagnosis and a positive serologic or molecular SARS-CoV-2 test result. The clinical and demographic characteristics of patients are summarized overall and by outcome in Table 1. Overall, 147 (48%) were identified as male and 118 (29%) were older adults (≥65 years old). The patient race/ethnicity distribution included 104 (34%) non-Hispanic (NH) white, 21 (7%) NH Black patients, 126 (42%) Hispanic, 27 (9%) Asian, and 25 (8%) other/unknown. The primary language for a subset 71 (23%) of patients was Spanish. The insurance distribution for the study cohort was 75 (25%) Medicaid, 104 (34%) Medicare, 95 (31%) commercial, or other 29 (10%). A total of 106 (35%) had a body mass index (BMI) ≥30 and 182 (60%) had ≥2 co-morbidities. The baseline chronic diseases among the study cohort included pulmonary disease n = 71 (23%), cardiovascular disease n = 150 (50%), renal disease n = 39 (13%), and autoimmune disease n = 13 (4%).

The majority of patients had solid tumors (n = 240, 79%) while the remaining had malignant hematologic cancers (n = 63, 21%). The cancer type distribution is summarized in the supplementary Table 1. Among the study cohort, at the time of COVID-19 diagnosis, 140 (46%) patients were in remission, 80 (26%) had stable disease or response on current therapy, and 54 (18%) had documented cancer progression on most recent clinical evaluation in the medical record. A total of 84 (28%) patients were considered immunosuppressed at time of COVID-19 diagnosis. The majority (n = 171, 56%) of patients had prior surgery and a smaller subset had prior radiation therapy (n = 77, 25%). A large proportion of patients (n = 207, 68%) had prior systemic therapy including myelosuppressive therapy (n = 128, 42%), hormone therapy (n = 48, 16%), and targeted therapy (n = 61, 20%).

### COVID-19 Presentation Characteristics

As shown in Table 2 the initial COVID-19 presentation characteristics varied by organ-specific complications. The majority of patients presented afebrile (n = 166, 55%), with active cough (n = 176, 58%), symptomatic (n = 204, 67%), and without a known COVID-19 exposure (n = 265, 88%). A subset (n = 22, 7%) had asymptomatic screening testing. Among the 137 (45%) patients who presented with fever, 68 (50%) had complications overall, 19 (14%) had sepsis, 56 (41%) had pulmonary complications, and 28 (20%) had cardiac complications. Among the 176 (58%) patients with cough, 90 (51%) had any complication, 22 (13%) had sepsis, 73 (42%) had pulmonary complication, and 44 (25%) had cardiac complications. The frequency of presentation varied over time with the peak presentation occurring in July 2021 (n = 71, 23%).

### Multivariate Logistic Regression

As shown in Table 3, patients with malignant hematologic cancers had a significantly higher risk of sepsis (OR 3.93 (95% CI 1.58–9.81)). Male patients had a greater risk of sepsis (OR 4.42 (95% CI 1.63–11.96)) and cardiac complications (OR 2.02 (95% CI 1.05–3.89)). There was a clear association between race/ethnicity and the risk of complications. Specifically, Hispanic patients had a higher risk of any complication (OR 2.31 (95% CI 1.18–4.51)) and other minority race group (non-Hispanics and non-whites) was associated with a higher odds of cardiac complications (OR 2.41 (95% CI 1.01–5.73)). Clinically, fever, cough, and ≥2 co-morbidities were independently significantly associated with any complication. Having ≥2 co-morbidities was also observed to be significantly associated with sepsis (OR 4.16 (95% CI 1.24–13.96)) and pulmonary complications (OR 2.48 (95% CI 1.27–4.84)). The effect of facility level COVID-19 7 day prior positivity rate was not significant in the model.

## Discussion

This study suggests that the clinical and demographic characteristics of cancer patients at time of initial COVID-19 presentation affects the likelihood of subsequent complications. In this analysis, Hispanic patients were observed to have twice the risk of developing any complication from COVID-19 compared to NH white patients. This increased risk has also been observed on a population level. As of July 2021, the Centers for Disease Control reports that Hispanic persons have twice the rate of COVID-19 and almost three times the rate of hospitalization due to COVID-19 compared to NH white persons [14]. The unique risks among Hispanic cancer patients with COVID-19 observed in this study confirmed previous reports by Grivas and colleagues who observed that Hispanic ethnicity was associated with higher COVID-19 illness severity compared to NH white patients [15].

This study also observed that male patients had four times the risk of sepsis after COVID-19 infection. A “sex-bias” in COVID-19 illness severity and mortality has previously been reported [12]. In a meta-analysis published by Desai and colleagues, cancer subtype (hematologic versus solid) and male sex were identified as factors that contribute to higher mortality estimates for hospitalized patients with cancer and COVID-19 [15]. In a pooled analysis of 2968 cancer patients, male sex was identified as having a higher risk of severe illness and death attributable to COVID-19 [17]. Sex-based differences in sepsis outcomes have also previously been reported, with women having a significantly better prognosis in the setting of sepsis [18]. These observations suggest that the previously reported increased COVID-19 associated mortality among male patients may be driven by an increased risk for sepsis after COVID-19 infection. In this study cohort, 21 (7%) patients had deaths attributed to COVID-19, though mortality was found to be associated with an active cancer diagnosis and advanced age (65+) rather than male sex [13]. A future analysis with a larger sample size will need to further examine this association.

Other clinical factors were shown to predict risk for complications such as multi-morbidity (≥2 co-morbidities) which was observed to be independently significantly associated with any complication, sepsis, and pulmonary complications. The notion that multimorbidity leads to adverse outcomes at time of COVID-19 infection has also been suggested by prior research. Moey and colleagues identified that patients with cancer and comorbid cardiovascular disease are at an increased risk of COVID-19 associated mortality [19]. The COVID-19 and Cancer Consortium (CCC19) study identified cardiovascular and pulmonary comorbidities to predict higher risk of COVID-19 severity among cancer patients with active disease [15]. Similarly, Borno and colleagues reported that patients in this study cohort with multi-morbidity had higher odds of hospitalization (citation).

Additionally, this analysis demonstrated that unique clinical presentation factors such as fever and cough may be associated with organ-specific complications. A systematic review and meta-analysis of 148 COVID-19 research studies identified fever and cough as the most common symptoms among adults infected with SARS-CoV2 [19]. The present study observed that symptoms of fever independently predict a higher odds of any complications and pulmonary complications. Robilotti and colleagues have previously reported that fever and cough are associated with a higher risk of hospitalization and severe respiratory illness among cancer patients [21]. Moreover, presence of cough in this study cohort was identified as a predictor of ICU hospitalization [13].

This study’s primary strength is that it leverages an ethnically diverse cohort to identify risk factors for COVID19 complications. Evaluating predictors of independent complications begins to provide a mechanistic understanding of differences in mortality previously reported among cancer patients with a COVID-19 diagnosis. The primary weakness of this analysis is its retrospective nature and limited sample size. Tumor types were grouped as solid or malignant hematologic cancers, therefore unique risk factors by disease type could not be elucidated in this dataset. Additionally, this study has limited follow-up therefore did not evaluate long-term complications of COVID-19 among cancer patients. Despite these limitations, to our knowledge this is the first analysis to evaluate multiple organ-specific complications among a diverse patient population.

## Conclusions

This analysis evaluated covariates that can significantly predict a myriad of complications among a multi-ethnic cohort of cancer patients. The conclusions drawn from this analysis elucidate a mechanistic understanding of differential illness severity from COVID-19. Observations from this analysis may help inform mitigation strategies to reduce adverse outcomes among minority populations. Future research will evaluate complications by tumor type and identify risk factors for long-term complications from COVID-19 among cancer patients.

**Table 1:** Patient characteristics

	Total			COVID-19 Outcomes															
				Any complications				Sepsis				Pulmonary complications				Cardiac complications			
	**N**	row %	column %	0		1		0		1		0		1		0		1	
				**N**	row %	**N**	row%	**N**	row %	**N**	row %	**N**	row %	**N**	row %	**N**	row %	**N**	row %
Total	303	100	100.0	184	60.7	119	39.3	273	90.1	30	9.9	212	70	91	30	246	81.2	57	18.8
Facility																			
UC San Diego Health	139	100	45.9	90	64.7	49	35.3	133	95.7	6	4.3	108	77.7	31	22.3	121	87.1	18	12.9
UC San Francisco Health	164	100	54.1	94	57.3	70	42.7	140	85.4	24	14.6	104	63.4	60	36.6	125	76.2	39	23.8
Gender																			
Female	156	100	51.5	96	61.5	60	38.5	149	95.5	7	4.5	113	72.4	43	27.6	134	85.9	22	14.1
Male	147	100	48.5	88	59.9	59	40.1	124	84.4	23	15.6	99	67.3	48	32.7	112	76.2	35	23.8
Age																			
< 65	185	100	61.1	118	63.8	67	36.2	169	91.4	16	8.6	135	73	50	27	155	83.8	30	16.2
>=65–100	118	100	38.9	66	55.9	52	44.1	104	88.1	14	11.9	77	65.3	41	34.7	91	77.1	27	22.9
Race																			
Non-Hispanic (NH) White	104	100	34.3	73	70.2	31	29.8	98	94.2	6	5.8	79	76	25	24	89	85.6	15	14.4
NH Black	21	100	6.9	12	57.1	9	42.9	17	81	4	19	15	71.4	6	28.6	16	76.2	5	23.8
Hispanic/Latinx	126	100	41.6	70	55.6	56	44.4	115	91.3	11	8.7	84	66.7	42	33.3	104	82.5	22	17.5
Asian	27	100	8.9	13	48.1	14	51.9	21	77.8	6	22.2	16	59.3	11	40.7	18	66.7	9	33.3
Other/Unknown	25	100	8.3	16	64	9	36	22	88	3	12	18	72	7	28	19	76	6	24
BMI																			
0 to 30	197	100	65.0	121	61.4	76	38.6	179	90.9	18	9.1	141	71.6	56	28.4	164	83.2	33	16.8
>=30	106	100	35.0	63	59.4	43	40.6	94	88.7	12	11.3	71	67	35	33	82	77.4	24	22.6
Insurance																			
Medicaid	75	100	24.8	43	57.3	32	42.7	66	88	9	12	51	68	24	32	62	82.7	13	17.3
Medicare	104	100	34.3	52	50	52	50	87	83.7	17	16.3	64	61.5	40	38.5	76	73.1	28	26.9
Commercial	95	100	31.4	69	72.6	26	27.4	91	95.8	4	4.2	77	81.1	18	18.9	84	88.4	11	11.6
Other	29	100	9.6	20	69	9	31	29	100			20	69	9	31	24	82.8	5	17.2
Cancer type																			
Solid Tumor	240	100	79.2	150	62.5	90	37.5	224	93.3	16	6.7	174	72.5	66	27.5	197	82.1	43	17.9
Malignant Hematologic Cancer	63	100	20.8	34	54	29	46	49	77.8	14	22.2	38	60.3	25	39.7	49	77.8	14	22.2
Smoking history																			
Never Smoker	210	100	69.3	131	62.4	79	37.6	195	92.9	15	7.1	152	72.4	58	27.6	176	83.8	34	16.2
Prior or current smoker	93	100	30.7	53	57	40	43	78	83.9	15	16.1	60	64.5	33	35.5	70	75.3	23	24.7
Cancer status																			
Unknown	29	100	9.6	16	55.2	13	44.8	24	82.8	5	17.2	17	58.6	12	41.4	21	72.4	8	27.6
Remission/no evidence of disease	140	100	46.2	86	61.4	54	38.6	130	92.9	10	7.1	101	72.1	39	27.9	117	83.6	23	16.4
Active disease with response/stable disease	80	100	26.4	13	61.9	8	38.1	18	85.7	3	14.3	15	71.4	6	28.6	16	76.2	5	23.8
Active disease with disease progression	54	100	17.8	37	62.7	22	37.3	54	91.5	5	8.5	41	69.5	18	30.5	49	83.1	10	16.9
Influenza vaccine status																			
Not vaccinated	94	100	31.0	57	60.6	37	39.4	85	90.4	9	9.6	65	69.1	29	30.9	76	80.9	18	19.1
Prior vaccination	143	100	47.2	87	60.8	56	39.2	128	89.5	15	10.5	104	72.7	39	27.3	121	84.6	22	15.4
Unknown	66	100	21.8	40	60.6	26	39.4	60	90.9	6	9.1	43	65.2	23	34.8	49	74.2	17	25.8
Primary language																			
English	219	100	72.3	145	66.2	74	33.8	199	90.9	20	9.1	163	74.4	56	25.6	184	84	35	16
Spanish	71	100	23.4	35	49.3	36	50.7	64	90.1	7	9.9	42	59.2	29	40.8	55	77.5	16	22.5
Other	13	100	4.3	4	30.8	9	69.2	10	76.9	3	23.1	7	53.8	6	46.2	7	53.8	6	46.2
Marital status																			
Single/Legally separated/Divorced/Widowed	124	100	40.9	70	56.5	54	43.5	111	88.7	13	10.5	86	69.4	38	30.6	103	83.1	21	16.9
Married/In relationship/Significant other	172	100	56.8	108	62.8	64	37.2	155	90.1	17	9.9	120	69.8	52	30.2	137	79.7	35	20.3
Unknown/Declined	7	100	2.3	6	85.7	1	14.3	7	100			6	85.7	1	14.3	6	85.7	1	14.3
Employment status																			
Unemployed	132	100	43.6	64	48.5	68	51.5	109	82.6	23	17.4	76	57.6	56	42.4	99	75	33	25
Currently employed	79	100	26.1	57	72.2	22	27.8	76	96.2	3	3.8	65	82.3	14	17.7	68	86.1	11	13.9
Unknown	92	100	30.4	63	68.5	29	31.5	88	95.7	4	4.3	71	77.2	21	22.8	79	85.9	13	14.1
Immunosuppressed status																			
Not immunosuppressed	219	100	72.3	137	62.6	82	37.4	202	92.2	17	7.8	158	72.1	61	27.9	177	80.8	42	19.2
Immunosuppressed	84	100	27.7	47	56	37	44	71	84.5	13	15.5	54	64.3	30	35.7	69	82.1	15	17.9
History of pulmonary disease																			
Not present	232	100	76.6	148	63.8	84	36.2	211	90.9	21	9.1	172	74.1	60	25.9	194	83.6	38	16.4
Present	71	100	23.4	36	50.7	35	49.3	62	87.3	9	12.7	40	56.3	31	43.7	52	73.2	19	26.8
History of cardiovascular disease																			
Not present	153	100	50.5	102	66.7	51	33.3	141	92.2	12	7.8	117	76.5	36	23.5	129	84.3	24	15.7
Present	150	100	49.5	82	54.7	68	45.3	132	88	18	12	95	63.3	55	36.7	117	78	33	22
History of renal disease																			
Not present	264	100	87.1	164	62.1	100	37.9	243	92	21	8	186	70.5	78	29.5	215	81.4	49	18.6
Present	39	100	12.9	20	51.3	19	48.7	30	76.9	9	23.1	26	66.7	13	33.3	31	79.5	8	20.5
Autoimmune disease																			
Not present	290	100	95.7	177	61	113	39	261	90	29	10	205	70.7	85	29.3	236	81.4	54	18.6
Present	13	100	4.3	7	53.8	6	46.2	12	92.3	1	7.7	7	53.8	6	46.2	10	76.9	3	23.1
Co-morbidity																			
0 to 1	121	100	39.9	86	71.1	35	28.9	117	96.7	4	3.3	99	81.8	22	18.2	105	86.8	16	13.2
>=2	182	100	60.1	98	53.8	84	46.2	156	85.7	26	14.3	113	62.1	69	37.9	141	77.5	41	22.5
Concomitant medications																			
0	26	100	8.6	16	61.5	10	38.5	22	84.6	4	15.4	20	76.9	6	23.1	21	80.8	5	19.2
1 to 3	159	100	52.5	108	67.9	51	32.1	150	94.3	9	5.7	125	78.6	34	21.4	137	86.2	22	13.8
4 to 6	106	100	35.0	56	52.8	50	47.2	91	85.8	15	14.2	62	58.5	44	41.5	79	74.5	27	25.5
7+	12	100	4.0	4	33.3	8	66.7	10	83.3	2	16.7	5	41.7	7	58.3	9	75	3	25
Concomitant medications																			
Corticosteroids	53	100	17.5	25	47.2	28	52.8	46	86.8	7	13.2	27	50.9	26	49.1	36	67.9	17	32.1
ACE inhibitors	45	100	14.9	26	57.8	19	42.2	42	93.3	3	6.7	30	66.7	15	33.3	37	82.2	8	17.8
Angiotensin receptor blockers	32	100	10.6	25	78.1	7	21.9	30	93.8	2	6.3	25	78.1	7	21.9	28	87.5	4	12.5
Anti-virals	58	100	19.1	32	55.2	26	44.8	48	82.8	10	17.2	36	62.1	22	37.9	43	74.1	15	25.9
Aspirin	54	100	17.8	38	70.4	16	29.6	48	88.9	6	11.1	39	72.2	15	27.8	44	81.5	10	18.5
Ibuprofen, naproxen, or other NSAIDs	70	100	23.1	42	60	28	40	64	91.4	6	8.6	49	70	21	30	57	81.4	13	18.6
Treatment history																			
Surgical Treatment	171	100	56.4	110	64.3	61	35.7	162	94.7	9	5.3	126	73.7	45	26.3	144	84.2	27	15.8
Radiation Therapy	77	100	25.4	46	59.7	31	40.3	68	88.3	9	11.7	53	68.8	24	31.2	65	84.4	12	15.6
Systemic Therapy	207	100	68.3	126	60.9	81	39.1	185	89.4	22	10.6	144	69.6	63	30.4	168	81.2	39	18.8
Myelosuppressive Therapy	128	100	42.2	76	59.4	52	40.6	116	90.6	12	9.4	88	68.8	40	31.3	106	82.8	22	17.2
Hormone Therapy	48	100	15.8	33	68.8	15	31.3	45	93.8	3	6.3	36	75	12	25	37	77.1	11	22.9
Targeted Therapy	61	100	20.1	31	50.8	30	49.2	48	78.7	13	21.3	37	60.7	24	39.3	48	78.7	13	21.3

**Table 2:** COVID-19 Presentation characteristics

	Total			COVID-19 Outcomes															
				Any complications				Sepsis				Pulmonary complications				Cardiac complications			
				0		1		0		1		0		1		0		1	
	**N**	row %	column %	**N**	row %	**N**	row %	**N**	row %	**N**	row %	**N**	row %	**N**	row %	**N**	row %	**N**	row %
Total	303	100	100.0	184	60.7	119	39.3	273	90.1	30	9.9	212	70	91	30	246	81.2	57	18.8
Fever																			
Afebrile	166	100	54.8	115	69.3	51	30.7	155	93.4	11	6.6	131	78.9	35	21.1	137	82.5	29	17.5
Fever	137	100	45.2	69	50.4	68	49.6	118	86.1	19	13.9	81	59.1	56	40.9	109	79.6	28	20.4
Cough																			
No cough	127	100	41.9	98	77.2	29	22.8	119	93.7	8	6.3	109	85.8	18	14.2	114	89.8	13	10.2
Cough	176	100	58.1	86	48.9	90	51.1	154	87.5	22	12.5	103	58.5	73	41.5	132	75	44	25
Month of confirmed infection (2020)																			
March	25	100	8.3	11	44	14	56	20	80	5	20	14	56	11	44	17	68	8	32
April	21	100	6.9	13	61.9	8	38.1	19	90.5	2	9.5	14	66.7	7	33.3	17	81	4	19
May	25	100	8.3	16	64	9	36	25	100			18	72	7	28	21	84	4	16
June	51	100	16.8	28	54.9	23	45.1	43	84.3	8	15.7	35	68.6	16	31.4	39	76.5	12	23.5
July	71	100	23.4	50	70.4	21	29.6	65	91.5	6	8.5	53	74.6	18	25.4	61	85.9	10	14.1
August	56	100	18.5	33	58.9	23	41.1	51	91.1	5	8.9	38	67.9	18	32.1	44	78.6	12	21.4
September	32	100	10.6	23	71.9	9	28.1	29	90.6	3	9.4	25	78.1	7	21.9	28	87.5	4	12.5
October	19	100	6.3	10	52.6	9	47.4	18	94.7	1	5.3	14	73.7	5	26.3	17	89.5	2	10.5
November	3	100	1.0			3	100	3	100			1	33.3	2	66.7	2	66.7	1	33.3
Symptomatic infection																			
Asymptomatic	99	100	32.7	77	77.8	22	22.2	92	92.9	7	7.1	80	80.8	19	19.2	85	85.9	14	14.1
Symptomatic	204	100	67.3	107	52.5	97	47.5	181	88.7	23	11.3	132	64.7	72	35.3	161	78.9	43	21.1
Known exposure																			
Not known	265	100	87.5	159	60	106	40	235	88.7	30	11.3	183	69.1	82	30.9	211	79.6	54	20.4
Known	38	100	12.5	25	65.8	13	34.2	38	100			29	76.3	9	23.7	35	92.1	3	7.9

**Table 3.** Multivariate logistic regression to evaluate factors that increase odds of COVID-19 complications

Covariates	Odds ratio (OR) (95% confidence interval (CI))			
	Any complications	Sepsis	Pulmonary complications	Cardiac complications
	Events N=119(39.3%)	Events N=30(9.9%)	Events N=91(30%)	Events N=57(18.8%)
Malignant hematologic cancer	1.09 ( 0.56–2.13 ) 0.8054	**3.93 ( 1.58–9.81 ) 0.0033**	1.53 ( 0.76–3.10 ) 0.2362	1.10 ( 0.50–2.42 ) 0.8079
Active cancer	0.82 ( 0.46–1.45 ) 0.4893	0.74 ( 0.30–1.84 ) 0.5132	0.73 ( 0.39–1.36 ) 0.3228	1.06 ( 0.54–2.08 ) 0.8672
Male	1.03 ( 0.60–1.78 ) 0.9165	**4.42 ( 1.63–11.96 ) 0.0034**	1.27 ( 0.70–2.28 ) 0.4333	**2.02 ( 1.05–3.89 ) 0.0354**
Hispanic/Latinx	**2.31 ( 1.18–4.51 ) 0.0143**	1.94 ( 0.57–6.58 ) 0.2893	1.96 ( 0.94–4.08 ) 0.0707	1.59 ( 0.69–3.71 ) 0.2786
Other/Unknown Race	1.86 ( 0.88–3.89 ) 0.1023	**4.43 ( 1.32–14.86 ) 0.0161**	1.47 ( 0.66–3.28 ) 0.3509	**2.41 ( 1.01–5.73 ) 0.0468**
Age 65+	1.43 ( 0.78–2.61 ) 0.2452	0.87 ( 0.34–2.23 ) 0.7693	1.50 ( 0.79–2.86 ) 0.2190	1.36 ( 0.67–2.77 ) 0.3954
BMI 30+	0.96 ( 0.54–1.71 ) 0.8940	1.50 ( 0.58–3.88 ) 0.4070	1.19 ( 0.64–2.21 ) 0.5917	1.92 ( 0.95–3.87 ) 0.0678
Fever	**1.88 ( 1.05–3.39 ) 0.0345**	1.96 ( 0.75–5.11 ) 0.1667	**2.20 ( 1.16–4.18 ) 0.0157**	0.78 ( 0.38–1.61 ) 0.5073
Cough	**2.71 ( 1.48–4.96 ) 0.0012**	1.39 ( 0.50–3.84 ) 0.5293	**3.23 ( 1.62–6.42 ) 0.0008**	**2.89 ( 1.35–6.19 ) 0.0062**
2+ Comorbidities	**1.80 ( 1.00–3.26 ) 0.0503**	**4.16 ( 1.24–13.96 ) 0.0211**	**2.48 ( 1.27–4.84 ) 0.0077**	1.17 ( 0.56–2.41 ) 0.6801

All emboldened text are statistically significant results
